# A Controlled-Release Nanofertilizer Improves Tomato Growth and Minimizes Nitrogen Consumption

**DOI:** 10.3390/plants12101978

**Published:** 2023-05-15

**Authors:** Mohamed I. D. Helal, Mohamed M. El-Mogy, Hassan A. Khater, Muhammad A. Fathy, Fatma E. Ibrahim, Yuncong C. Li, Zhaohui Tong, Karima F. Abdelgawad

**Affiliations:** 1 Soil Sciences Department, Faculty of Agriculture, Cairo University, Giza 12613, Egypt; 2 Vegetable Crops Department, Faculty of Agriculture, Cairo University, Giza 12613, Egypt; 3 Department of Soil and Water Sciences, Tropical Research and Education Center, Institute of Food and Agricultural Science (IFAS), University of Florida, Homestead, FL 33031, USA; 4 School of Chemistry and Bimolecular Engineering, Georgia Institute of Technology, Atlanta, GA 30332, USA

**Keywords:** *Solanum lycopersicum*, quality, nitrogen use efficiency, urea, nanoparticles, ecofriendly

## Abstract

Minimizing the consumption of agrochemicals, particularly nitrogen, is the ultimate goal for achieving sustainable agricultural production with low cost and high economic and environmental returns. The use of biopolymers instead of petroleum-based synthetic polymers for CRFs can significantly improve the sustainability of crop production since biopolymers are biodegradable and not harmful to soil quality. Lignin is one of the most abundant biopolymers that naturally exist.In this study, controlled-release fertilizers were developed using a biobased nanocomposite of lignin and bentonite clay mineral as a coating material for urea to increase nitrogen use efficiency. Five types of controlled-release urea (CRU) were prepared using two ratios of modified bentonite as well as techniques. The efficiency of the five controlled-release nano-urea (CRU) fertilizers in improving the growth of tomato plants was studied under field conditions. The CRU was applied to the tomato plants at three N levels representing 100, 50, and 25% of the recommended dose of conventional urea. The results showed that all CRU treatments at the three N levels significantly enhanced plant growth parameters, including plant height, number of leaves, fresh weight, and dry weight, compared to the control. Additionally, most CRU fertilizers increased total yield and fruit characteristics (weight, length, and diameter) compared to the control. Additionally, marketable yield was improved by CRU fertilizers. Fruit firmness and acidity of CRU treatments at 25 and 50% N levels were much higher than both the 100% CRU treatment and the control. The vitamin C values of all CRU treatments were lower than the control. Nitrogen uptake efficiencies (NUpE) of CRU treatments were 47–88%, which is significantly higher than that of the control (33%). In conclusion, all CRU treatments at an N level of 25% of the recommended dose showed better plant growth, yield, and fruit quality of tomatoes than the conventional fertilizer.

## 1. Introduction

The world population is predicted to exceed 9.7 billion by 2064 [1]. Current agricultural practices cannot meet rising food demand without heavy fertilizer use. However, conventional fertilizers are inherently limited due to low nutrient use efficiency (NUE) (30–35% for nitrogen (N), 18–20% for phosphorus (P), and 35–40% for potassium (K)) [2]. Low NUEs inevitably lead to increased fertilizer use in order to maintain agricultural yields. The cost of energy and materials associated with this strategy increases the financial burden on farmers and impedes the development of sustainable agriculture. In addition, heavy fertilizer use contributes to various environmental problems, such as greenhouse gas emissions and water quality deterioration [3].

Nitrogen plays an essential role in plant growth, crop yield, and quality [4]. To sustain high crop yield in most arable soils, a high quantity of N fertilizer has been used, but nitrogen use efficiency has been decreasing even in developing countries [5,6]. The low NUE of urea is usually the result of the rapid dissolution and/or transformation of nutrients into forms unavailable to plants. Therefore, there is great interest in developing innovative fertilizers to increase NUEs. Controlled-release fertilizers (CRFs) have been developed by coating core soluble fertilizers with materials (sulfur or polymer) that limit the exposure of core fertilizers to water and release nutrients by diffusion [7]. This brings out the idea of developing encapsulated fertilizers, in which NPK fertilizers are entrapped within nanoparticles [8]. Nanomaterial surface coatings on fertilizer particles make them more strongly adherent due to higher surface tension than conventional surfaces, allowing for gradual release [9]. Given the unique properties of nanomaterials, incorporating nanotechnology into the design and using innovative fertilizers is a strategy with great potential [10,11]. 

The use of nanofertilizers is the most important application of nanotechnology in agriculture [12]. Some properties of nanoparticles, including large specific surface area, unique magnetic/optical properties, electronic states, and catalytic reactivity, give nanoparticles better reactivity than their bulk counterparts [12]. Many previous works studied the effect of nanofertilizers on the growth and production of crops such as green pepper [13], and green beans [14]. Davarpanah et al. [15] reported that pomegranate fruit yield was improved similarly with two applications each of nano-nitrogen fertilizer and urea at rates of 1.8 and 16.3 kg N/ha, respectively. 

Tomato (*Solanum lycopersicum L.*) is an important crop due to its economic [16] and nutritional importance [17]. According to our knowledge, rare studies have looked into the effect of nano-urea slow/controlled-release fertilizer on tomato production. Additionally, the urea encapsulation and producing CRU used in this study are biodegradable and not harmful to soil quality, compared with petroleum-based synthetic polymers used for slow-release fertilizers (SRFs). The aim of this study was to evaluate the efficiency of nano-ureacontrolled-release fertilizer (CRU) on tomato plant growth, production, quality, and nitrogen use efficiency.

## 2. Materials and Methods

### 2.1. Preparation of Nano-Urea Controlled-Release Fertilizer (CRU)

A method of the three major steps, as described by Zhang et al. (2020) [18], was used with two main modifications to further slowdown the nitrogen release rate. These are the sequential addition of urea solution and sodium alginate and using a higher modified bentonite suspension of 5%, instead of 2%, to increase the thickness and hardness of the coating, increasing the residence time in soil. The three steps of preparation are:

(a) Synthesis of quaternary ammonium lignin (QAL): In an ice-salt bath (NaCl/ice = 1:3 *w/w*), trimethylamine (TMA) and epichlorohydrin were mixed at a molar ratio of 10:7, then left overnight for the complete reaction and formation of epoxypropyl trimethylammonium chloride (ETAC). The ETAC was added to a lignin solution prepared by dissolving 2.5 g of lignin into 25 mL of 20 wt.% NaOH. The mixture was stirred for 5 h at 80 °C until obtaining brown-red emulsion. The obtained product (QAL) was then dried under a vacuum.
(b) Preparation of QAL—modified bentonite clay mineral. A 5 g amount of bentonite clay mineral was added to 400 mL of deionized water (DW) and stirred until a homogenous suspension was obtained. A suspension containing 10 g QAL and 100 mL DW was added to the bentonite clay mineral suspension and stirred overnight. The modified bentonite was separated from the aqueous phase by centrifugation at 3500 rpm, then washed several times with DW and later ethanol. After that, the product was freeze-dried and stored in a refrigerator at 4 °C.
(c) Preparation of nano-ureacontrolled-release fertilizer (CRU). In the third step, the CRU was formed by encapsulating urea with QAL-modified bentonite in the presence of sodium alginate and calcium chloride using different techniques. Suspensions of 2 and 5 wt.% of QAL-modified bentonite were prepared and stirred until stability and homogeneity, then a saturated urea solution was added to the suspension and stirred overnight. After that, the mixture was heated to 80°C and 2 wt.% of sodium alginate solution was added and stirred to form a gel. The formed gel was kept in a refrigerator for 24 h for stabilization, then dropped into a heated 4% calcium chloride solution to form the beads of CRU. The beads were separated, then dried in an oven at 55 °C.

The five types of CRU differed in the percentages of N and coating thickness. The percentages of N were 17.5, 24.5, 22.8, 27.3, and 23.5% for T1, T2, T3, T4, and T5, respectively. The second difference is due to the sequential addition of sodium alginate and urea solution to the QAL-modified bentonite, and to the concentration of QAL-modified bentonite suspension used in the preparation. In the original method, sodium alginate was initially added before the urea solution, so the bentonite interlayer penetration of urea was incomplete. Additionally, a suspension of 2 and 5 wt.% of QAL-modified bentonite was used, instead of using 2% in the original method. Except for the beads of T1, urea was initially added before sodium alginate, so the bentonite interlayer penetration of urea was complete for the latter ones compared with the former one. For the beads of T1, T2, and T4, 2% *w*/*v* QAL-modified bentonite suspension was used, whereas the 5% ones were used for preparing the beads of T3 and T5.

### 2.2. Plant Growth Conditions

Seeds of tomato plants, hybrid GS010 (from Syngenta company, Basel, Switzerland), were sown in foam trays (209 wells) filled with peat moss and vermiculite media (1:1, *v*:*v*) in a greenhouse (9 m wide × 40 m long × 2.5 m high) on 15 November 2019. After 45 days, seedlings were transferred to 6 L black plastic pots containing 5 kg of clay loamy soil. The basic properties of soil used in this study were presented in Table 1. The soil is alluvial, the order is Entisols, and the great group is Typic Torrifluvent. All pots received 150 kg ha^−1^ P_2_O_5_ as triple super phosphate before transplanting and 200 kg ha^−1^ K_2_O as potassium sulfate, of which 50 kg ha^−1^ was applied at 15 days and 75 kg ha^−1^ each at 40 and 60 days after transplanting. The quantity of commercial urea (N = 46%)/pot (the pot contains 5 kg soil) = 1.3 gm, which represents the full dose (100%). This quantity was split into three doses of 0.543, 0.433, and 0.324 gm added at 15, 40, and 60 days of transplanting. For the other treatments, a quantity of fertilizer containing similar N units was calculated based on the N% in each treatment of T1–T5 and according to the N level (100, 50, and 25%).

Irrigation water with 7.25 pH and 0.42 dSm^−1^ was applied regularly to maintain soil moisture at 75% of the field capacity. Disease and pest control were managed according to traditional practices. The mean relative humidity during plant growth ranged between 65 and 75%, and the average temperature was 25/15 °C (day/night). The photon flux density was typically between 800 and 1000 µmol m^−2^ s^−2^.

### 2.3. Nitrogen Fertilizer Treatments

A randomized complete block design (RCBD) with two factors was used for this study. Five types (T1, T2, T3, T4, and T5) of CRU plus a control (commercial urea of 100% of the recommended dose). Three levels (100, 50, and 25%) of N were applied for all five types of CRU treatments. Six replicates for each treatment were used. Total content at a rate of 300 kg N ha^−1^ was applied for the control and 100% N level of all treatments of CRU by splitting into three doses of 125, 100, and 75 kg Nha^−1^ at 15, 40, and 60 days after transplanting, respectively.

### 2.4. Measurements of Plant Growth, Fruit Yield, and Quality

Plant height, number of leaves, stem diameter, number of branches, and relative chlorophyll content were measured 15 days after the application of the final N dose. Fresh and dry weights of plants were measured at harvesting. To measure the plant height, the distance between the soil surface to the highest growing tip was measured. Chlorophyll content was measured by SPAD– 502 Chlorophyll meter (Konica-Minolta, Osaka, Japan). Both total yield, number of fruits per plant, and marketable yield (free from any physiological disorders) were recorded. In addition, 20 random fruits from each replicate were taken to measure the fruit length, diameter, and weight. The firmness value of each fruit was determined twice at two sites on opposite sides of the center of the fruits with a fruit pressure tester (FT011, Wagner Instruments, Milan, Italy), then the mean value was calculated. The total soluble solids content (TSS) of tomato fruits was measured using a digital refractometer (PR101, Palette, Co., Ltd., Tokyo, Japan) at 25 °C. The degrees Brix was determined by placing a few drops of the clear juice on the lens, then recording the degrees Brix°. Deionized water was used for calibration. The lens was rinsed between samples with deionized water.

Fruit acidity was measured by homogenizing tengrams of fresh fruit material in 100 mL of distilled water. Then, 10 mL of the aliquot was titrated with 0.1 N NaOH in the presence of phenolphthalein as an indicator. The acidity percent was calculated from the titration data as a percentage of citric acid in the juice [19]. Vitamin C was analyzed based on a titration method as described by Abdallah et al. [20]. Briefly, 10 g fresh fruits were homogenized with 90 mL of oxalic acid (6%) for 10 min., then 2,6-dichlorophenol indophenol was used to titrate 25 mL of filtrated solution. The results were expressed as mg 100 g^−1^ of fresh weight.

Leaf nitrogen and potassium: Portions of 0.2 g oven-dried (60–70 °C) tomato leaves were digested with sulfuric acid (98% *w*/*v*) and hydrogen peroxide (H_2_O_2_, 30%) according to the method of Estefan et al. [21], then N concentration was determined in the acid digestion extract using ammonia distilling unit of Kjeldahl (Protein-Nitrogen Distiller, RAYPA, Barcelona, Spain). Potassium concentration was determined in the acid digestion extract using flame emission spectrophotometry (Corning 4100, Corning, UK).

### 2.5. Measurements of Soil and Irrigation Water Properties

The properties determined for irrigation water and the composite soil sample represent a mixture of four individual ones collected from the soil used for packing the pots used for growing tomato plants are soil pH (measured directly in irrigation water and in 1:2.5 soil–water suspension using a pH meter) (Accumet AR.20, Fisher Scientific, Waltham, MA, USA). The electrical conductivity (EC) was measured in irrigation water and in the filtrate of the suspension of 1:2.5 soil–water ratio, using an EC meter (JENWAY, London, UK, 4510). The total carbonate content of the soil was determined by means of Collin’s calcimeter as described by Jackson [22] and calculated as calcium carbonate. Total organic carbon (OC) content was determined in the experimental soil using standard methods of Walkley and Black [23] based on wet oxidation by dichromate. This method is suitable for materials to contain relatively low organic matter contents. Plant-available nitrogen was extracted from the experimental soil using 2 M KCl solution, then the total N concentration was determined using an ammonia distilling unit of Kjeldahl (Protein-Nitrogen Distiller, RAYPA, Barcelona, Spain). Mechanical analysis was performed according to the pipette method as described by Gee and Bauder [24] and the class was obtained from the Texture Triangle.

Nitrogen uptake efficiency (NUpE) was calculated using the equation; N_f*_100/(N*_soil_* + N*_fert_*.), gg^−1^, where N*_f_*= final aboveground plant N amount at the end of the main growth period, N*_soil_* = soil N concentration (kg ha^−1^), and N*_fert_* = N amount fertilized (kg ha^−1^) according to Moll et al. [25]. The N level in soil represents the available N in soil (mineral N) that was determined by extracting the soil using 2 M KCl solution (at 1:5 soil: solution ratio, and shaking for 1 h). After filtration, the total soluble N (NO_3_^−^ and NH_4_^+^) was determined in the filtrate by an ammonia distilling unit in the presence of Devarda’s alloy, then calculated as kg N ha^−1^ [21].

### 2.6. Statistical Analyses

All obtained data were subjected to analysis of a two-way ANOVA test using MSTAT software (2.1 Michigan State University, East Lansing, MI, USA). The means were compared at the 95% probability level according to the Tukey test. The heat-map figures were created by SPSS software, version 21.

## 3. Results and Discussion

### 3.1. Plant Growth and Nutrient Uptake

**Figure 1:**
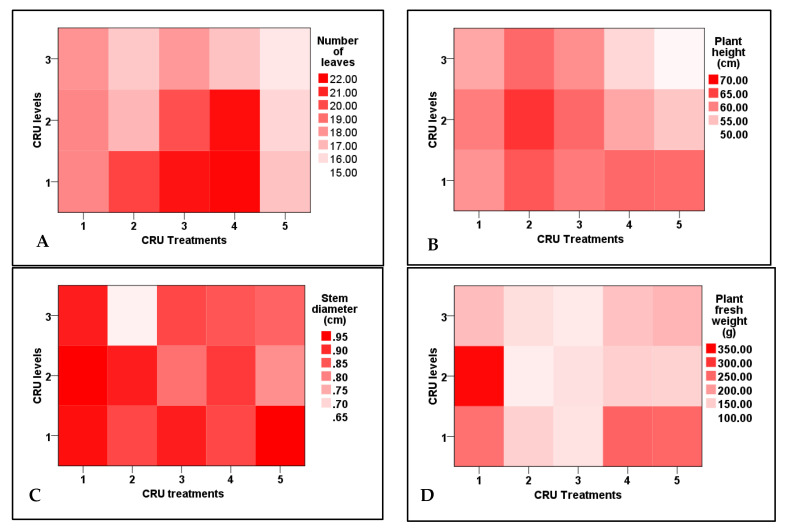
Two-dimensional heat-map visualization shows the interaction between the CRU treatments and applied N levels (100, 50, and 25% of the recommended dose) for plant growth attributes ((**A**): number of leaves, (**B**): plant height, (**C**): stem diameter, and (**D**): plant fresh weight).

All growth attributes (plant height, stem diameter, number of leaves and branches, fresh and dry weight) of tomato plants (Table 2) were significantly affected positively by treatments of CRU at all applied N levels (100, 50, and 25% of the recommended dose).The results in Table 2 show that all tomato growth parameters increased as the applied N level of CRU increased from 25 to 100% of the recommended dose. The number of branches was significantly increased for all treatments and N levels, but their interaction is insignificant (Table 2). Plant heights of all CRU treatments at all N levels were significantly higher than these of the control treatment (Table 3). Among all treatments, the plants of T2 at an N level of 50% were the tallest ones (Figure 1). This result could be due to the role of capsulated nano-urea in providing the N element during the whole growing season and conserving the N by absorbing the majority of the element [26]. On the other hand, the surface tension of nanomaterial-coated fertilizer particles is higher than that of conventional fertilizer particles; they are stronger and hence more effective at regulating the release of nutrients [27].

In all CRU treatments at the 100 and 50% N levels, the number of leaves and stem diameter were significantly higher than those of the lowest N level (25%). The highest number of leaves was recorded for T3 at an N level of 100% and T4 at 100 and 50% (Figure 1). The results of Table 1 and Table 2 indicated that the stem diameter of tomato plants of all treatments increased as the N level increased from 25% to 100%. The highest values of stem diameter were recorded for T1, T3, and T5 at an N level of 100% (Figure 1). In accordance with these results, Salimi et al. [28] found that the shoot fresh weight, shoot dry weight, root fresh weight, root dry weight, plant height, number of leaves, and number of branches of tomato seedlings plants increased by slow-release urea application compared to uncoated urea. Except for the plants of T2 at an N level of 50%, the fresh weights of all CRU treatments at all N levels are higher than the control. Among all treatments, the highest plant fresh weight was observed for T1 at 50% N level (Table 3 and Figure 1). Kazem et al. [29] obtained a similar result and observed an increase in tomato plant fresh weight by increasing N application rates.

**Figure 2:**
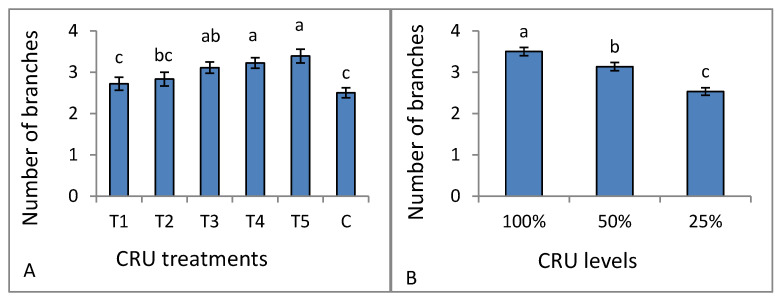
Number of branches of tomato plants as affected by CRU: (**A**) treatments and (**B**) applied N levels. Different letters indicate significant differences between the levels according to Tukey’s multiple range test (*p* ≤ 0.05).

The dry weight of all CRU treatments at all N levels was higher than that of the control (Table 3). An agreement with our result was reported by Fan and Li [30] and Elia and Conversa [31], who recorded that shoot dry weight increased by increasing the N fertilizer application rate. The CRU treatments of T5, T4, and T3 had a number of branches higher (without a significant difference) than T2, T1, and the control treatment (Figure 2A). All CRU treatments at N levels of 100 and 50% had a higher number of branches than 25% (Figure 2B). Generally, the observed increase in all growth parameters could be attributed to the fact that the N nutrient promotes tomato vegetative growth and development by enhancing photosynthesis resulting in higher biomass [32]. Our results are in agreement with Degefa et al. [33], who reported that the increase in tomato plant growth traits is related to the increase in N fertilizer. Generally, we can say that the observed increases in all traits could be attributed to the fact that nitrogen element is responsible for many important physiological processes in plants, including both photosynthesis and protein synthesis that lead to an increase in biomass. At the same time, its absence or deficiency reduces plant growth.

### 3.2. Biomass of Tomato Plants and Chemical Composition of Leaves

**Figure 3:**
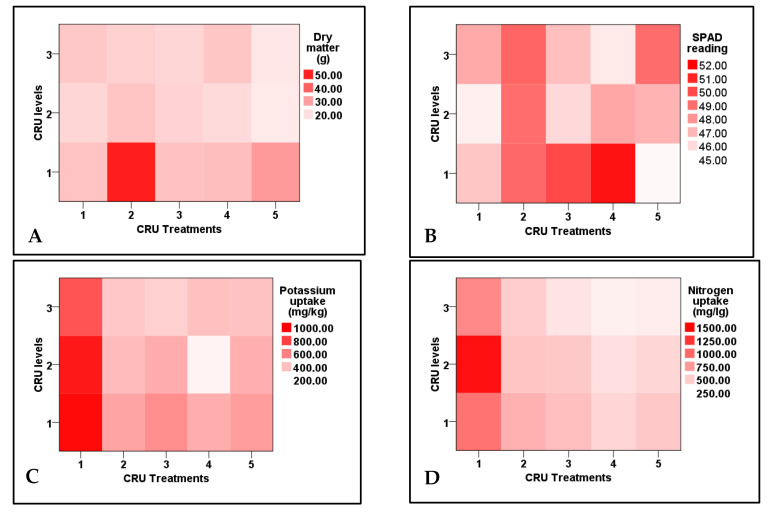
Two-dimensional heat-map visualization shows the interaction between CRU treatments and applied N levels (100, 50, and 25% of the recommended dose) for (**A**) dry matter, (**B**) SPAD reading, (**C**) potassium uptake, and (**D**) nitrogen uptake by tomato plants.

Data shown in Table 4indicate that plant dry matter, leaf chlorophyll (SPAD reading), potassium and nitrogen contents, and their uptake by tomato plants were highly significantly affected by CRU treatments, applied N levels, and their interaction. The dry matter contents of all CRU treatments at all N levels were higher than the control, except for T5 at 50 and 25% N levels. The highest dry matter contents were recorded for T2, followed by T5 at an N level of 100% compared with these for the control and other CRU treatment (Table 5 and Figure 3). Leaves relative chlorophyll (SPAD reading) of all CRU treatments was higher than that of the control (Table 5). Among all treatments, the highest SPAD values were recorded for the plants of T4 and T3 at an N level of 100% (Table 5 and Figure 3). Similar results were reported by Salimi et al. [28], who found that chlorophyll content in tomato leaves was increased by slow-release urea treatment, which could be attributed to the relation between chlorophyll synthesis and leaves N concentration. Most CRU treatments at most N levels significantly increased K and N uptake by tomato plants compared with control (Table 5. The highest K and N uptake values were obtained for T1 at all N levels of 100, 50, and 25% (Table 5 and Figure 3). Similar results were obtained by Salimi et al. [28], who reported a significant increase in N and K contents in tomato plants by the urea slow-release application compared to conventional urea.

### 3.3. Tomato Yield and Its Components

**Figure 4:**
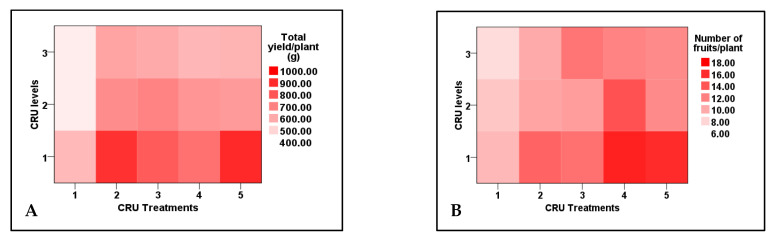

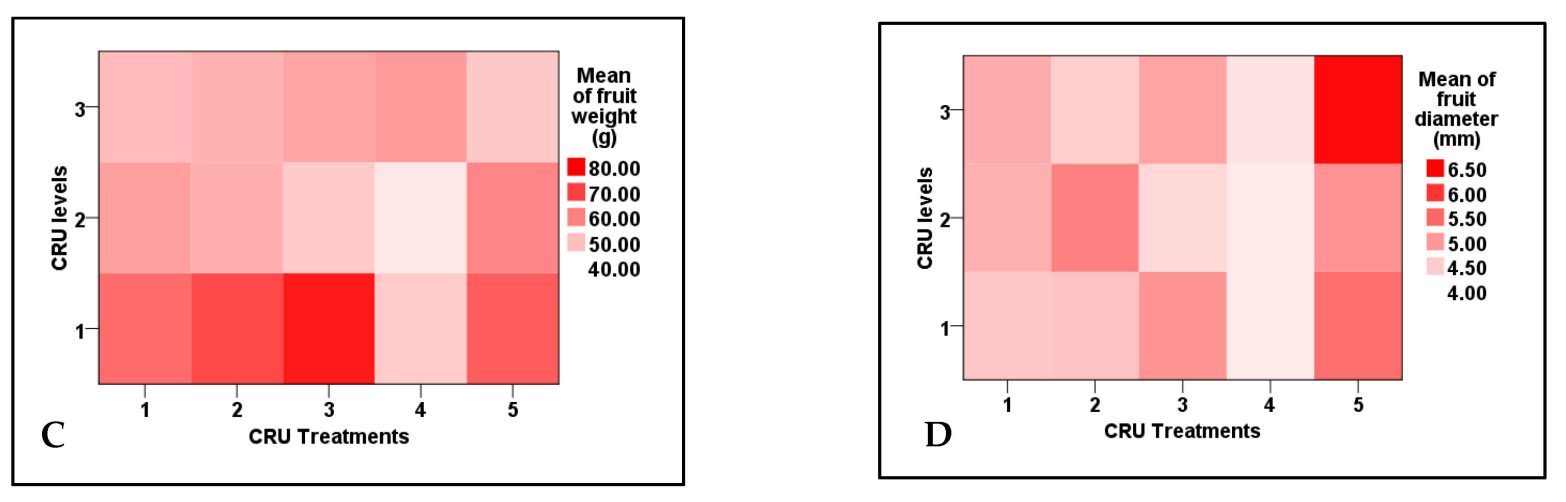
Two-dimensional heat-map visualization shows the interaction between the CRU treatments and applied N levels (100, 50, and 25% of the recommended dose) for (**A**) total yield/plant, (**B**), number of fruits/plant, (**C**) mean of fruit weight, and (**D**) mean of fruit diameter.

**Figure 5:**
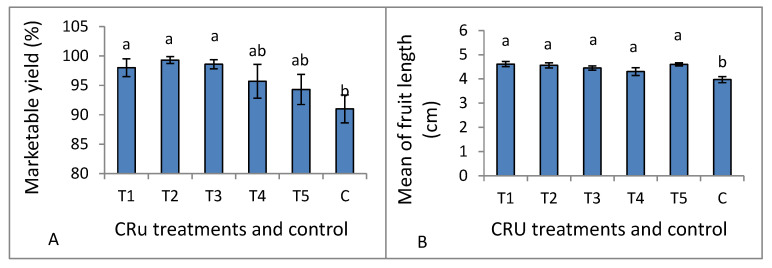
Marketable yield (**A**) and mean of fruit length (**B**) as affected by CRU treatments. Different letters indicate significant differences between the treatments according to Tukey’s multiple range test (*p* ≤ 0.05).

Data listed in Table 6 indicate that both total yield and the number of fruits per plant, mean of fruit weight, and mean of fruit diameter were significantly affected by CRU treatments, applied N levels, and their interaction, whereas marketable yield and mean of fruit length are affected significantly only by CRU treatments. The total tomato fruit yield/plant of all CRU treatments increased considerably as the N level increased from 25 to 100%, except for T1 at the 50 and 25% N levels. The total tomato fruit yield/plant of all CRU treatments for all N levels was significantly higher than those for the control (Table 6 and Table 7). The highest total yields per plant are recorded for T2 and T5 treatments at an N level of 100% (Figure 4). A previous study [34] indicated that the highest tomato yield was obtained for the plot treated with a higher N rate than the control. In accordance with our results, Davarpanah et al. [35] found that nano-urea application increased the fruit number of pomegranates compared to conventional urea. Additionally, Kinoshita et al. [36] reported that applying controlled-release fertilizers increased tomato yield compared to conventional fertilizers. The results of Table 7 indicate that the mean tomato fruit weight increased significantly as the N level increased from 25 to 100% for most CRU treatments. The highest mean of fruit weight values was recorded for T3, followed by T2 at an N level of 100% (Figure 4). The fruit diameter values of most CRU treatments were higher than the control, and the highest value is observed for T5 at the 25% N level (Figure 4). The marketable yield of T1, T2, and T3 are significantly higher than those of the control (Figure 5A). The mean values of tomato fruit length of all CRU treatments were higher than those of the control (Figure 5B). Our results are in agreement with those of Davarpanah et al. [35], who found that the pomegranate fruit length was increased by nano-urea application compared with traditional urea. Generally, the great enhancement in all yield parameters of tomato plants treated with CRU at all N levels could be attributed to their slow-release characteristics, which increase the residence time of fertilizer in soil and increase plant utilization efficiency, hence decreasing the nutrient loss [37]. The in vitro study of our team found that the release of N in soil from all CRU used in this study was sustained for one month. The important finding of these results is that, except for T1 treatments, the total tomato yields per plant of all CRU treatments at an N level of 25% are higher than the control (full dose of commercial urea). The consumption of nitrogenous fertilizers could be reduced to 25% of the recommended dose by using the CRU prepared in our lab.

### 3.4. Tomato Fruit Quality and Nitrogen Uptake Efficiency

Tomato fruit firmness and acidity, contents of ascorbic acid, and nitrogen uptake efficiency are significantly affected by CRU treatments, N applied levels, and their interactions. The highest TSS values were observed for T3 at the 50% N level (Supplementary Figure S1). Similar results were obtained by Davarpanah et al. [35], who reported that the TSS in pomegranate arils was significantly increased by nano N application. Additionally, TSS is not significantly affected by applied N levels, but affected by CRU types and their interaction with N levels (Table 8). Except for T5 at an N level of 25%, the TSS of all CRU treatments at all N levels are significantly higher, in most treatments, than the control (Table 9).

Among all treatments, including the control one, fruit firmness of T1 at 25 and T2 at 100% N levels was the lowest, whereas that of T1 and T5 at 100% N level was the highest (Supplementary Figure S1). The firmness of all other CRU treatments was insignificantly higher than the control (Table 8). Similar results were recorded by Wang et al. [38], who found that tomato fruit firmness initially increased with increasing N levels, then decreased at the highest N levels. Although the nitrogen element is required for the optimal firmness of tomato fruits, higher N levels reduce the movement of calcium, which leads to the lowness of firmness [39,40].

**Figure 6:**
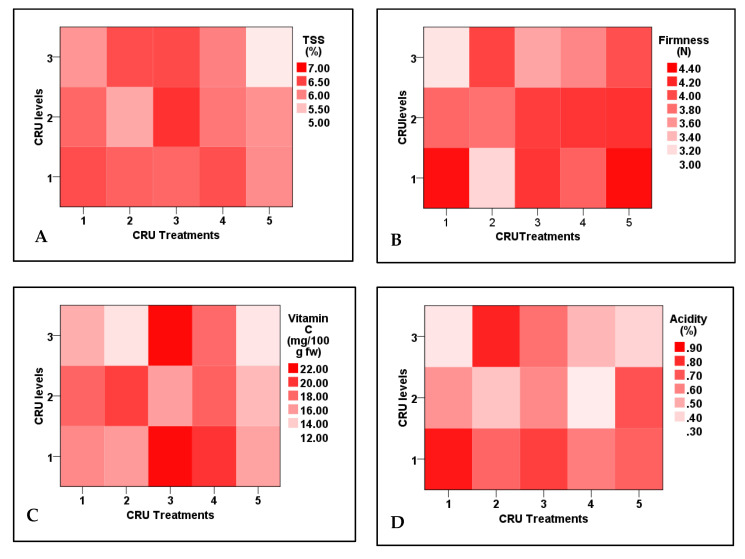
Two-dimensional heat-map visualization shows the interaction between CRU treatments and applied N levels (100, 50, and 25% of the recommended dose) for (**A**) TSS, (**B**) firmness, (**C**) vitamin C, and (**D**) acidity.

Data presented in Table 8 show that, except for T3 and T4 at N levels of 25 and 100%, which have the highest contents of vitamin C, their contents in the rest of CRU treatments at all N levels (100, 50, and 25%) were significantly lower than the control (Figure 6). Similar results were recorded by Zhang et al. [41] and Kuscu et al. [42]. They reported decreases in vitamin C content in tomato fruits by increasing N fertilization rate, which may be due to higher oxidative stress caused by high N levels [43]. Regarding fruit acidity, except for T1 and T4 at N levels of 25 and 50% and T2 and T5 at 50 and 25%, respectively, the acidity of all CRF treatments is significantly higher than the control (Table 9). The highest fruit acidity values were observed for T1 and T2 at 100% and 25%, respectively (Figure 6). Generally, the fruit acidity increased by increasing N levels. Similar results are reported by Wang et al. [38], who reported a significant increase in fruit acidity of cherry tomatoes with increasing N fertilization rate. The elevation in fruit acidity with the increase in N fertilization rate could be attributed to the increase in the synthesis of organic acids [44]. Generally, most CRU treatments at the applied N level of 25% enhanced fruit quality parameters over control one, so consumption of N fertilizers could be reduced to 25% of the recommended dose without any reduction in tomato fruit quality. Nitrogen uptake efficiency (NUpE) values of all CRU treatments were considerably higher and raised to be near one hundred percent (88%) compared with control one (33%). Except for T2, T3, T4, and T5 at an N level of 100%, those have NUpF values ranging from 47 to 49%. All CRU treatments at all N levels have NUpE values (52–88%) significantly higher than the control (33%). In a previous study, the NUpE was increased by CRU treatment of urea fertilizer [45]. Additionally, Saha et al. [46] found that compared to conventional urea fertilizer, the total nitrogen percentage that remained in the soil after SRF treatment was greater.

## 4. Conclusions

In this study, nanotechnology has been used to develop a CRU that releases nitrogen gradually in soil to match plant needs, reduce loss, and ultimately increase agricultural production. Our CRU is an environmentally friendly and cost-effective nanofertilizer. The materials, bentonite clay mineral and biorefinery lignin, used for urea encapsulation and producing the CRU are biodegradable and not harmful to soil quality, compared with petroleum-based synthetic polymers used for slow-release fertilizers (SRFs). These two materials are not expensive.Bentonite naturally occurs in mines worldwide, and many companies extract it in large quantities, and lignin is a byproduct of paper manufacturing and can also be produced from agricultural residues. Our CRU has high percentages of N ranging from 17.5 to 27.3%, indicating its significance to be used as a high-quality nitrogenous fertilizer to feed plants. Except for urea and ammonium nitrate, the percentages of N in the CRU are higher than all mineral nitrogenous fertilizers available in the agro-market. All these items represent a significant contribution to the research. Additionally, we evaluated the efficiency of five types of nano-ureacontrolled-release fertilizers (CRU) on tomato growth, fruit yield and quality, and plant nitrogen uptake efficiency. The results indicated that all five CRU significantly enhanced plant height, fresh and dry weight, stem diameter, and the number of leaves and branches compared to the control (full dose of commercial urea). The highest total yield, fruit number per plant, and fruit weight were recorded for 100% N levels of T2, T4, and T3 treatments, respectively. Firmness and fruit acidity were markedly enhanced by the lowest and moderate N levels (25 and 50%) of most treatments compared to the control. On the other hand, the vitamin C values of tomato fruits of all CRU treatments were lower than the control one. Nitrogen uptake efficiency (NUpE) of all CRU treatments was enhanced considerably and raised to be near one hundred percent (88%) compared with the control (33%). The important finding of this study is that the quantity and quality of the tomato yield of all CRU treatments at the 25% N level were significantly higher than the control (full dose of commercial urea; 300 kg Nha^−1^), which means that the consumption of nitrogenous fertilizers could be reduced to be 25% of the recommended dose by applying the CRU of the current research.

## Figures and Tables

**Table 1:** Chemical and physical properties of the soil used in the tomato pot experiment.

pH	EC (dS/m)	TCC * %	OC ** %	Available Nutrients (mg/kg)			Coarse Sand %	Fine Sand %	Silt %	Clay %	Tex. Class
				N	P	K					
7.58	2.8	2.7	1.65	111.4	13.5	220	4.4	31.0	27.3	37.3	Clay Loam

* Total calcium carbonate, ** total organic carbon.

**Table 2:** Analysis of variance of tomato growth attributes.

Source	df	Plant Height (cm)	Number of Branches	Number of Leaves	Stem Diameter (mm)	Plant Fresh Weight (g)	Plant Dry Weight (g)
Treatments (T)	4	575.9 ***	2.02 ***	35.43 ***	0.08 ***	45786.75 ***	2764.71 ***
Levels (L)	3	156 ***	5.95 ***	48.50 ***	0.04 *	17363.17 ***	366.98 ***
T × L	12	39.96 *	0.27 ^ns^	9.77 **	0.01 *	13886.22 ***	236.61 ***

ns: not significant. *, **, ***: significant at *p* ≤ 0.05, ≤ 0.01, and ≤ 0.001, respectively.

**Table 3:** Plant growth attributes as affected by CRU treatments x applied N levels interaction. CRU beads contain varied quantities of N, 25, 50, and 100% of recommended dose. The percentages of N were 17.5, 24.5, 22.8, 27.3, and 23.5%, for T1, T2, T3, T4, and T5, respectively.

Treatment	Level	Plant Height (cm)	Number of Leaves	Stem Diameter (mm)	Fresh Weight (g)	Dry Weight (g)
T1	100%	58.50 b–e *	18.33 bc	0.93 a	239.50 b	44.48 b
	50%	60.17 bc	18.33 bc	0.95 a	342.83 a	69.71 a
	25%	57.00 c–e	18.00 b–d	0.92 ab	165.83 cd	42.81 b
T2	100%	63.17 ab	20.17 ab	0.87 a–c	146.33 c–e	27.85 cd
	50%	66.00 a	17.00 cd	0.92 ab	117.83 e	25.28 c–f
	25%	61.83 a–c	16.50 cd	0.67 d	132.83 e	23.30 d–f
T3	100%	60.33 a–c	21.50 a	0.92 ab	128.50 e	29.60 c
	50%	61.83 a–c	19.83 ab	0.82 a–c	130.17 e	30.09 c
	25%	58.83 b–d	17.83 b–d	0.87 a–c	121.50 e	22.82 d–f
T4	100%	61.83 a–c	21.83 a	0.87 a–c	254.33 b	24.40 d–f
	50%	57.00 c–e	21.67 a	0.88 ab	147.00 c–e	20.15 fg
	25%	53.17 ef	16.67 cd	0.85 a–c	161.17 cd	22.05 ef
T5	100%	61.67 a–c	16.67 cd	0.95 a	248.83 b	26.30 c–e
	50%	54.50 d–f	16.17 cd	0.78 b–d	144.00 de	26.87 c–e
	25%	50.67 fg	15.67 d	0.83 a–c	172.67 c	22.82 d–f
Control		47.00 g	18.17 b–d	0.73 cd	119.83 e	16.64 g

* Means followed by a letter in common in the same column are not significantly different at 0.05 level of probability according to Tukey’s multiple range test (*n*= 6).

**Table 4:** Analysis of variance of tomato plants dry matter, leaf chlorophyll, and nitrogen and potassium content and uptake.

Source	df	Plant Dry Matter (%)	Leaf Chlorophyll (SPAD)	Potassium Uptake (mg/kg)	NitrogenUptake (mg/kg)	Potassium Content (%)	Nitrogen Content (%)
Treatments (T)	4	321.68 ***	59.25 ***	576850.164 ***	1267121.5 ***	0.083 ns	0.858 ns
Levels (L)	3	742.73 ***	13.87 **	422384.546 ***	164155.74 ***	0.722 **	5.420 **
T × L	12	219.37 ***	19.13 **	82231.437 ***	127315.57 ***	0.347 *	2.939 **

ns: not significant. *, **, ***: significant at *p* ≤ 0.05, ≤ 0.01, and ≤ 0.001, respectively.

**Table 5:** Tomato plants dry matter, leaf chlorophyll, and nitrogen and potassium uptake and content, as affected by CRU treatments x N levels interaction. CRU beads contain varied quantities of N, 25, 50, and 100% of recommended dose. The percentages of N were 17.5, 24.5, 22.8, 27.3, and 23.5%, for T1, T2, T3, T4, and T5, respectively.

Treatment	Levels	Plant Dry Matter (%)	LeafChlorophyll (SPAD)	Potassium Uptake (mg/kg)	Nitrogen Uptake (mg/kg)	Potassium Content (%)	Nitrogen Content (%)
T1	100%	24.42 cd *	46.57 c–f	972.12 a	938.51 b	2.20 a	2.10 a–c
	50%	21.42 c–e	45.45 ef	925.64 a	1429.12 a	1.33 de	2.05 a–d
	25%	23.60 cd	47.35 b–e	735.61 b	833.62 bco	1.73 a–d	1.94 a–e
T2	100%	50.45 a	49.13 a–d	484.82 c–e	507.13 de	1.72 a–d	1.81 b–e
	50%	24.30 cd	49 a–d	489.45 c–e	536.65 de	1.92 ab	2.10 a–c
	25%	22.38 c–e	49.22 a–c	376.21 d–g	469.51 d–f	1.63 b–e	2.02 a–e
T3	100%	24.80 cd	50.02 ab	452.51 c–f	561.81 d	1.53 b–e	1.90 a–e
	50%	21.97 c–e	46.05 d–f	533.64 cd	703.41 c	1.77 a–d	2.17 ab
	25%	21.38 c–e	46.75 c–f	347.87 e–g	381.88 e–g	1.51 b–e	1.67 de
T4	100%	25.17 c	51.52 a	454.32 c–f	457.21 d–g	1.87 a–c	1.88 b–e
	50%	20.82 c–e	47.42 b–e	436.41 e–g	346.52 fg	1.17 e	1.71 c–e
	25%	24.02 cd	45.62 ef	401.21 c–f	435.16 d–g	1.79 a–d	1.96 a–e
T5	100%	30.80 b	45.18 ef	536.05 c	505.70 de	2.01 ab	1.96 a–e
	50%	18.45 e	47.10 b–e	366.12 e–g	442.41 d–g	1.36 c–e	1.65 e
	25%	18.70 e	49.03 a–d	434.47 c–f	532.73 de	1.85 a–d	2.28 a
Control		20.18 de	43.85 f	309.91 fg	303.24 g	1.80 a–d	1.72 c–e

* Means followed by a letter in common in the same column are not significantly different at 0.05 level of probability according to Tukey’s multiple range test (*n* = 6).

**Table 6:** Analysis of variance of tomato yield attributes.

Source	df	Total Yield/ Plant (g)	Marketable Yield (%)	Number of Fruits/Plant	Mean of Fruits Weight (g)	Mean of Fruits	Mean of Fruits
						Diameter (cm)	Length (cm)
Treatments (T)	4	1499295.80 ***	174.32 *	82.43 ***	947.25 ***	4.04 ***	1.123 ***
Levels (L)	3	606361.64 ***	136.74 ^ns^	60.84 ***	1233.72 ***	0.428 *	0.102 ^ns^
T × L	12	237796.43 ***	31.32 ^ns^	8.83 *	288.55 ***	0.865 *	0.291 ^ns^

ns: not significant. *, ***: significant at *p* ≤ 0.05 and ≤ 0.001, respectively.

**Table 7:** Tomato yield attributes as affected by CRU treatments x applied N levels interaction. CRU beads contain varied quantities of N, 25, 50, and 100% of recommended dose. The percentages of N were 17.5, 24.5, 22.8, 27.3, and 23.5%, for T1, T2, T3, T4, and T5, respectively.

Treatment	Levels	Total Yield/	Number of	Mean of Fruits	Mean of Fruits
		Plant (g)	Fruits/Plant	Weight (g)	Diameter (cm)
T1	100%	564.95 f *	9.33 gh	63.13 b–d	4.55 b–d
	50%	443.06 g	8.67 gh	55.13 d–f	4.77 b–d
	25%	444.31 g	7.67 h	50.70 e–g	4.82 b–d
T2	100%	884.22 a	13.33 cd	68.33 ab	4.60 b–d
	50%	671.67 c–e	10.33 fg	52.58 e–g	5.25 bc
	25%	615.37 d–f	10.00 fg	52.07 e–g	4.48 cd
T3	100%	785.44 b	12.67 c–e	75.95 a	5.05 b–d
	50%	692.67 cd	10.67 e–g	48.27 f–h	4.38 cd
	25%	600.77 ef	12.50 c–e	54.25 ef	4.90 b–d
T4	100%	734.20 bc	16.50 a	47.95 f–h	4.22 d
	50%	649.72 c–f	14.17 bc	43.73 gh	4.22 d
	25%	570.72 f	11.83 d–f	55.72 d–f	4.28 d
T5	100%	902.55 a	16.00 ab	65.43 bc	4.42 b
	50%	638.03 d–f	11.50 d–f	58.93 c–e	5.07 b–d
	25%	575.58 f	11.50 d–f	48.63 f–h	5.40 d
Control		418.06 g	9.33 gh	40.63 h	4.45 cd

* Means followed by a letter in common in the same column are not significantly different at 0.05 level of probability according to Tukey’s multiple range test (*n* = 6).

**Table 8:** Analysis of variance of tomato fruit quality attributes and nitrogen uptake efficiency.

Source	df	TSS (Brix)	Firmness (N)	Vitamin C (mg/100 g)	Acidity (%)	NUpE
Treatments (T)	4	2.83 **	0.722 **	71.750 ***	0.077 ***	5173.91 ***
Levels (L)	3	0.434 ns	0.837 *	20.535 ***	0.258 ***	2519.71 ***
T × L	12	0.493 *	0.688 ***	28.321 ***	0.126 ***	430.57 ***

ns: not significant. *, **, ***: significant at *p* ≤ 0.05, ≤ 0.01, and ≤ 0.001, respectively.

**Table 9:** Tomato fruit quality attributes and nitrogen uptake efficiency as affected by CRU treatments x applied N levels interaction. CRU beads contain varied quantities of N, 25, 50, and 100% of recommended dose. The percentages of N were 17.5, 24.5, 22.8, 27.3, and 23.5%, for T1, T2, T3, T4, and T5, respectively.

Treatment	Level	TSS (Brix°)	Firmness (N)	Vitamin C (mg/100 g FW)	Acidity (%)	NUpE (%)
T1	100%	6.4 ab *	4.3 a	16.6 ef	0.84 a	83 a
	50%	6.2 a–c	3.8 a–c	18.0 d	0.55 gh	88 a
	25%	5.8 b–d	3.1 d	15.1 g	0.36 jk	80 ab
T2	100%	6.2 a–c	3.2 cd	15.9 fg	0.66 d–e	49 e
	50%	5.6 c–e	3.7 a–c	19.5 bc	0.44 ij	71 b–d
	25%	6.4 ab	4.0 ab	13.1 h	0.82 ab	69 cd
T3	100%	6.2 a–c	4.1 ab	16.68 fg	0.75 bc	48 e
	50%	6.6 a	4.0 ab	15.8 fg	0.57 fg	85 a
	25%	6.4 ab	3.5 b–d	21.6 a	0.63 d–g	80 a–c
T4	100%	6.3 ab	3.8 ab	20.0 b	0.60 e–g	47 e
	50%	6.0 a–c	4.1 ab	18.2 cd	0.34 k	52 e
	25%	6.0 a–c	3.6 b–d	17.8 de	0.47 hi	63 d
T5	100%	5.9 b–d	4.3 a	15.6 fgo	0.76 c–e	48 e
	50%	5.8 b–d	4.1 ab	14.7 g	0.70 cd	66 d
	25%	5.1 e	3.9 ab	13.0 h	0.40 i–k	70 cd
Control		5.3 de	3.5 b–d	18.9 b–d	0.55 jh	33 f

* Means followed by a letter in common in the same column are not significantly different at 0.05 level of probability according to Tukey’s multiple range test (*n* = 6).

## Data Availability

Not applicable.

## Supplementary Materials

The following supporting information can be downloaded at: https://www.mdpi.com/article/10.3390/plants12101978/s1, Figure S1. Tomato plants at the harvest stage of control and the five CRU treatments at N applied level of 25 (A), 50 (B), and 100%(C) of the recommended dose.
