# Supplementary material for: A Controlled-Release Nanofertilizer Improves Tomato Growth and Minimizes Nitrogen Consumption
Source: Plants (Basel). 2023 May 15;12(10):1978. doi: 10.3390/plants12101978 (PMC10223464; doi:10.3390/plants12101978)
Supplement: Supplementary file 1 [file plants-12-01978-s001.zip › plants-2286958-supplementary.pdf]

Supplementary figure

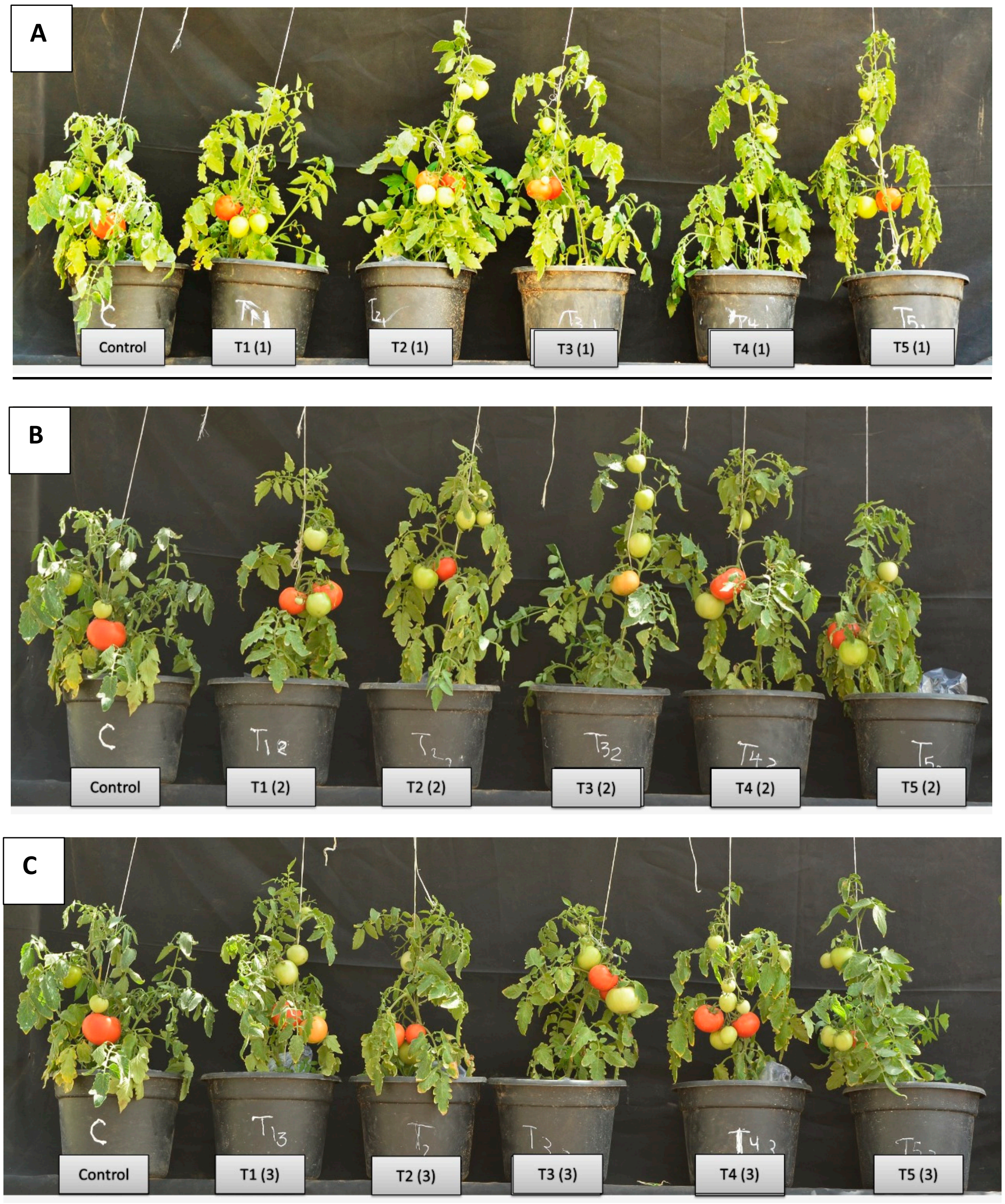

Figure S1. Tomato plants at the harvest stage of control and the five CRU treatments at N applied level of 25 (**A**), 50 (**B**), and 100%(**C**) of the recommended dose.
